# Proteolytic Activity of Human Lymphoid Tumor Cells. Correlation with Tumor Progression

**DOI:** 10.1155/2000/74372

**Published:** 2000

**Authors:** Angelo Vacca, Domenico Ribatti, Roberto Ria, Antonio Pellegrino, Michele Bruno, Francesca Merchionne, Franco Dammacco

**Affiliations:** 1 Department of Biomedical Sciences and Human Oncology Section of Internal Medicine and Clinical Oncology Bari 1-70124 Italy; 2 lnstitute of Human Anatomy, Histology and Embryology University of Bari Medical School Bari 1-70124 Italy; 3 Theodor Kocher Institute University of Bern Bern CH-3000 Swaziland; 4 Department of Biomedical Sciences and Human Oncology Section of Internal Medicine and Clinical Oncology Policlinico Piazza Giulio Cesare, 11 BARI I-70124 Italy

**Keywords:** Human lymphoid tumors, matrix-degrading enzymes, multiple myeloma, mycosis fungoides, proteolysis, tumor progression

## Abstract

Matrix metalloproteinase (MMP) expression and production are associated with advanced-stage tumor and contribute to tumor progression, invasion and metastases. The current study was designed to determine the expression and production of MMP-2 (gelatinase A) and MMP-9 (gelatinase B) by human lymphoid tumor cells. Changes in expression and production were also investigated during tumor progression of multiple myeloma and mycosis fungoides. *In situ* hybridization analysis revealed that lymphoblastic leukemia B cells (SB cell line), multiple myeloma (MM) cells (U266 cell line) and lymphoblastic leukemia T cells (CEM and Jurkat cell lines) express constitutively the mRNA for MMP-2 and/or MMP-9. We demonstrated by gelatin-zymography of cell culture medium that both enzymes were secreted in their cleaved (activated) form. *In situ* hybridization of bone marrow plasma cells and gelatin- zymography of the medium showed that patients with active MM (diagnosis, relapse, leukemic progression) express higher levels of MMP-2 mRNA and protein than patients with non-active MM (complete/objective response, plateau) and with monoclonal gammopathies of undetermined significance (MGUS). MMP-9 expression and secretion was similar in all patient groups. In patients with mycosis fungoides (MF), the expression of MMP-2 and MMP-9 mRNAs was significantly upregulated with advancing stage, in terms of lesions both positive for one of two mRNAs and with the greatest intensity of expression. Besides MF cells, the MMP-2 and/or MMP-9 mRNAs were expressed by some stromal cell populations (microvascular endothelial cells, fibroblasts, macrophages), suggesting that these cells cooperate in the process of tumor invasion. Our studies identify MMPs as an important class of proteinases involved in the extracellular matrix (ECM) degradation by human lymphoid tumors, and suggest that MMPs inhibitors may lead to important new treatment for their control.


*Developmental Immunology*, 2000, Vol. 7(2-4), pp. 77-88
Reprints available directly from the publisher
Photocopying permitted by license only

© 2000 OPA (Overseas Publishers Association)
N.V. Published by license under the
Harwood Academic Publishers imprint,
part of the Gordon and Breach Publishing Group.
Printed in Malaysia

### Proteolytic Activity of Human Lymphoid Tumor Cells. Correlation with Tumor Progression

ANGELO VACCA[a*], DOMENICO RIBATTI[b], ROBERTO RIA[a], ANTONIO PELLEGRINO[c], MICHELE BRUNO[a], FRANCESCA MERCHIONNE[a] and FRANCO DAMMACCO[a]

[a]*Department of Biomedical Sciences and Human Oncology, Section of Internal Medicine and Clinical Oncology and* [b]*Institute of Human Anatomy, Histology and Embryology, University of Bari Medical School, 1–70124 Bari, Italy and* [c]*Theodor Kocher Institute, University of Bern, CH-3000 Bern, Switzerland*

Matrix metalloproteinase (MMP) expression and production are associated with advanced-stage tumor and contribute to tumor progression, invasion and metastases. The current study was designed to determine the expression and production of MMP-2 (gelatinase A) and MMP-9 (gelatinase B) by human lymphoid tumor cells. Changes in expression and production were also investigated during tumor progression of multiple myeloma and mycosis fungoides. *In situ* hybridization analysis revealed that lymphoblastic leukemia B cells (SB cell line), multiple myeloma (MM) cells (U266 cell line) and lymphoblastic leukemia T cells (CEM and Jurkat cell lines) express constitutively the mRNA for MMP-2 and/or MMP-9. We demonstrated by gelatin-zymography of cell culture medium that both enzymes were secreted in their cleaved (activated) form. *In situ* hybridization of bone marrow plasma cells and gelatin-zymography of the medium showed that patients with active MM (diagnosis, relapse, leukemic progression) express higher levels of MMP-2 mRNA and protein than patients with non-active MM (complete/objective response, plateau) and with monoclonal gammopathies of undetermined significance (MGUS). MMP-9 expression and secretion was similar in all patient groups. In patients with mycosis fungoides (MF), the expression of MMP-2 and MMP-9 mRNAs was significantly upregulated with advancing stage, in terms of lesions both positive for one of two mRNAs and with the greatest intensity of expression. Besides MF cells, the MMP-2 and/or MMP-9 mRNAs were expressed by some stromal cell populations (microvascular endothelial cells, fibroblasts, macrophages), suggesting that these cells cooperate in the process of tumor invasion. Our studies identify MMPs as an important class of proteinases involved in the extracellular matrix (ECM) degradation by human lymphoid tumors, and suggest that MMPs inhibitors may lead to important new treatment for their control.

*Keywords:* Human lymphoid tumors, matrix-degrading enzymes, multiple myeloma, mycosis fungoides, proteolysis, tumor progression

* Correspondence to: Prof. Angelo VACCA, M.D., Department of Biomedical Sciences and Human Oncology, Section of Internal Medicine and Clinical Oncology, Policlinico, Piazza Giulio Cesare, 11, I-70124 BARI (Italy). Phone (39)-80–5478.826, Fax (39)-80–5478.820, E-mail avacca@hotmail.com

### INTRODUCTION

Many enzyme systems are involved in extracellular proteolytic events. Enzymes belong to one of two families, namely the serine proteases – in particular plasminogen activator (PA)/plasmin enzymes – and the matrix metalloproteinases (MMPs) (Mignatti and Rifkin, 1993). MMPs are a family of closely related zinc-endopeptidases whose principal function is the degradation of extracellular matrix (ECM) components during normal embryo development, morphogenesis and tissue remodelling. They are secreted as zymogen (activated extracellularly), and divided into three subgroups based on the substrate preference: the interstitial collagenases, stromelysin and gelatinases (type IV collagenases), althought all of them recognize overlapping substrate specificity (Massova et al., 1998).

Type IV collagenases include type IV 72 kDa (MMP-2 or gelatinase A) and 92 kDa (MMP-9 or gelatinase B) enzymes. Both MMP-2 and MMP-9 facilitate invasion and metastases because they degrade type IV, V, VII and X collagens as well as fibronectin (Mignatti and Rifkin, 1993; Massova et al., 1998) which are major constituents of the interstitial stroma and the subendothelial basement membrane ECM. Studies in animal models clearly demonstrated a positive correlation between MMP expression, invasive behavior and metastatic potential (Krane, 1994; Coussens and Werb, 1996; Stetler-Stevenson et al., 1997; Kanayama et al. 1998).

MMPs production *in vivo* and *in vitro* is increased in response to angiogenic agents (Coussen and Werb, 1996). In some conditions, such as diabetic retinopathy, the involvement of proteinases may be confined to the process of angiogenesis (Folkman, 1995); in others, such as cancer (Johnson et al., 1998) and rheumatoid arthritis (Gordon et al., 1993; Bottomley et al., 1998), proteinase activity is thought to be an integral part of generalized tissue remodelling, as well as of the formation of new blood vessels. Inhibition of MMP results in decreased invasion and metastasis of malignant cell lines (Mignatti and Rifkin, 1993; Krane, 1994).

The expression of MMPs has been shown in hematologic cells including polymorphonuclear leukocytes, macrophages and lymphocytes. Polymorphonuclear leucocyte infiltration and microvascular injury are allmarks the countersign of tissue remodelling associated with multiple organ failure. These processes require the concerted action of various proteolytic enzymes, including MMPs (Machein and Conca, 1997; Amorino and Hoover, 1998). Lymphoid cell extravasation into perivascular tissues during inflammation involves transmigration through the endothelial cell layer and basement membrane, and employement of adhesion mechanisms prior to degradation of the ECM, which are similar to the process of extravasation adopted by metastatic cells (Weeks et al., 1993; Madri et al., 1996; Di Girolamo et al., 1998; Trocme et al., 1998).

In the present study, we investigated human lymphoid tumor cells of B-cell and T-cell lineage for their invasive potential in well-defined clinical models of tumor progression.

### RESULTS

### MMP-2 and MMP-9 Expression and Production by Human Lymphoid Tumor Cells

SB (B-cell lymphoblastic leukemia), U266 (multiple myeloma), CEM and Jurkat (T-cell lymphoblastic leukemia) cell lines were evaluated for expression of MMP-2 and MMP-9 mRNA by *in situ* hybridization (Fig. 1). As summarized in Table I, SB and U266 cells co-expressed the mRNAs. The signal intensity for both mRNAs was similar in the B-lymphoblastic leukemia cell line SB, while U266 cells showed a more intense MMP-2 mRNA-related signal than that of MMP-9. The T-cell lymphoblastic leukemia cell lines CEM and Jurkat expressed only MMP-2 mRNA. The mRNAs were activated, given the fact that the enzymes were secreted into the cell culture conditioned medium (CM) and detected by sodium dodecyl sulfate (SDS)-polyacrylamide gel electrophoresis (PAGE) gelatin-zymography (Fig 2). MMP-2 and

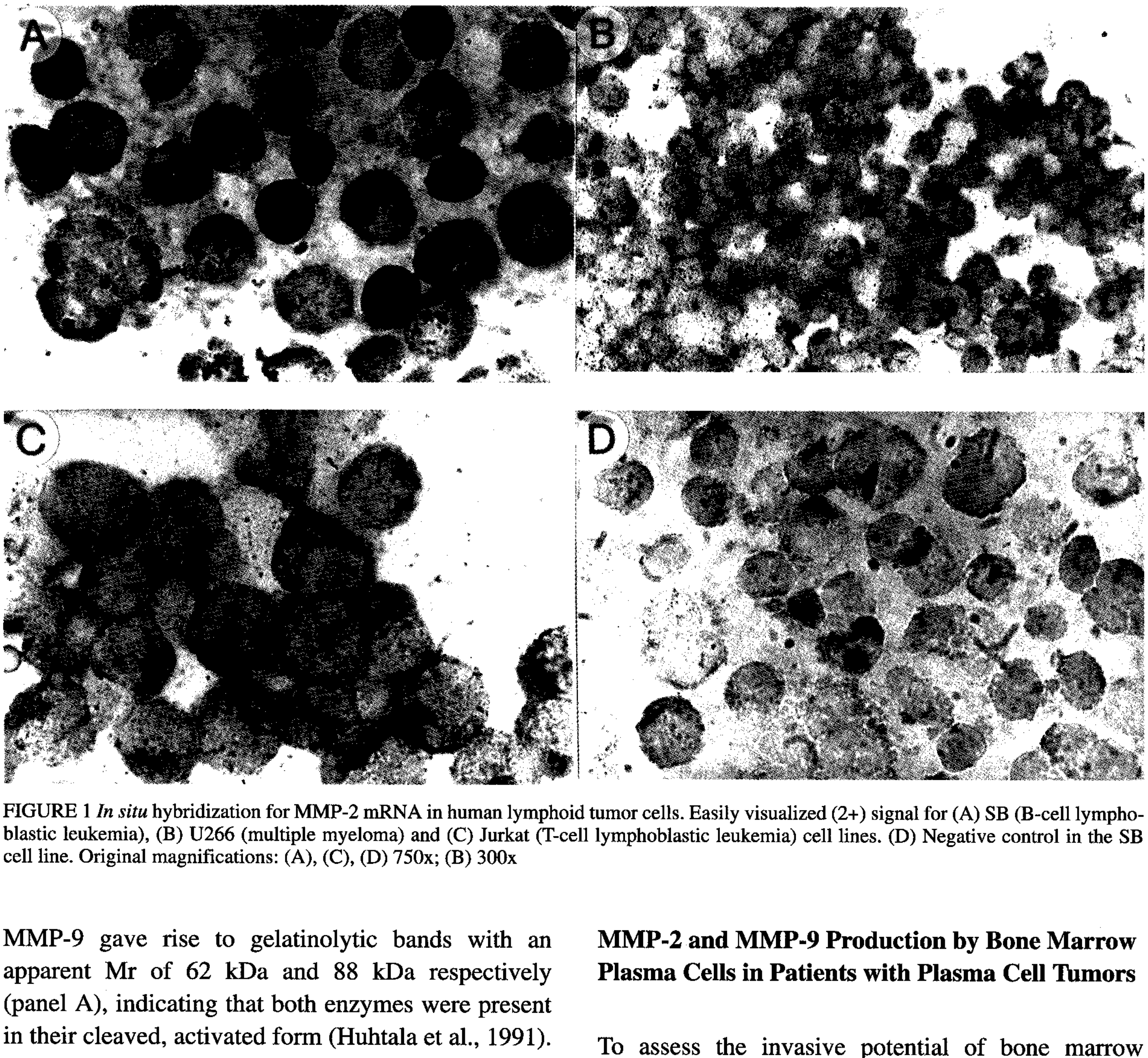

FIGURE 1 *In situ* hybridization for MMP-2 mRNA in human lymphoid tumor cells. Easily visualized (2+) signal for (A) SB (B-cell lymphoblastic leukemia), (B) U266 (multiple myeloma) and (C) Jurkat (T-cell lymphoblastic leukemia) cell lines. (D) Negative control in the SB cell line. Original magnifications: (A), (C), (D) 750x; (B) 300x

MMP-9 gave rise to gelatinolytic bands with an apparent Mr of 62 kDa and 88 kDa respectively (panel A), indicating that both enzymes were present in their cleaved, activated form (Huhtala et al., 1991). According to *in situ* hybridization pictures, the computerized image analysis of the bands (panel B) showed that overlapping amounts of MMP-2 and MMP-9 were present in the CM of SB cells, whereas MMP-2 levels were higher than those of MMP-9 in the CM of U266 cells, and the CM of CEM and Jurkat cells contained only MMP-2 gelatinolytic activity.

Overall results demonstrate that human lymphoid tumor cells constitutively produce significant amounts of the ECM-degrading enzymes MMP-9 and/or MMP-2, which are found in their cleaved, activated form.

### MMP-2 and MMP-9 Production by Bone Marrow Plasma Cells in Patients with Plasma Cell Tumors

To assess the invasive potential of bone marrow plasma cells during tumor progression, patients with plasma cell tumors at different progression stages were studied (Vacca et al., 1999), namely: *i*) patients with monoclonal gammopathies of under termined significance (MGUS); *ii*) patients with non-active multiple myeloma (MM), i.e. those in post-treatment complete/objective response or in the off-treatment plateau-phase; *iii*) patients with active MM (the progression stage), i.e. those at diagnosis with symptomatic disease and an increase in M-component level in the 3 months before analysis, or at relapse, or with unresponsive and rapidly progressive disease (leuke-

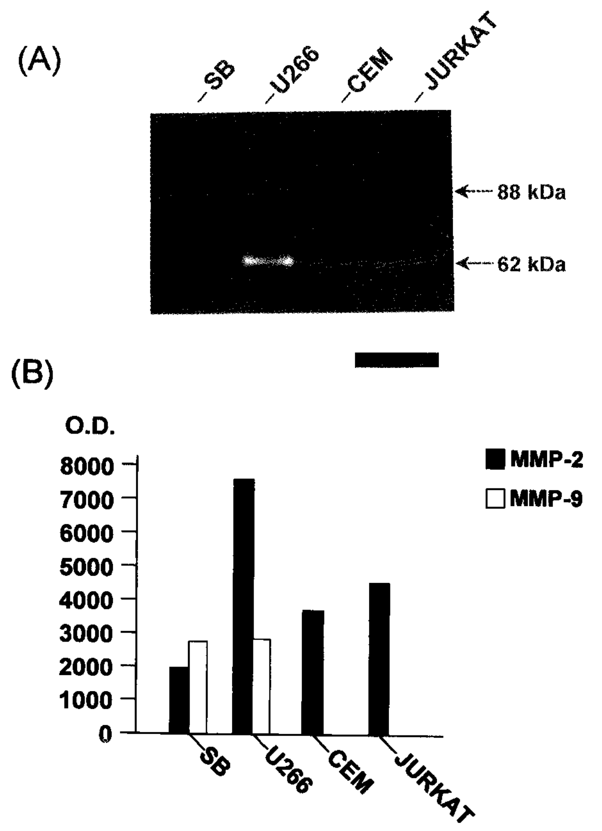

FIGURE 2 MMP-2 and MMP-9 secretion by human lymphoid tumor cells. (A) SDS-PAGE gelatin-zymography of the CM of SB, U266, CEM and Jurkat cell lines. Note the white bands against a dark background with an apparent Mr of 62 kDa and 88 kDa related to the gelatinolytic regions of activated (cleaved) MMP-2 and MMP-9 respectively. (B) quantitations of the intensity of the bands as optical density (O.D.), by using computerized image analysis mic progression). Control subjects were patients with benign anemia (due to iron or vitamin $B_{12}$ deficiency).

TABLE I *In situ* hybridization for MMP-2 and MMP-9 mRNA expression by human lymphoid tumor cell lines

| *mRNA of* | *Cell line* | | | |
|---|---|---|---|---|
| | *SB* | *U266* | *CEM* | *Jurkat* |
| MMP-2 | 2+[a] | 2+ | 2+ | 2+ |
| MMP-9 | 2+ | + | – | – |

a. (-), no signal; (+), detectable signal; (2+), easily visualized signal.

As shown in Fig. 3, bone marrow plasma cells expressed the MMP-2 and MMP-9 mRNA (panels A to G), whereas in plasma cells from a control subject the MMP-2 mRNA was absent (panel H) or poorly expressed in some instances, as evaluated by *in situ* hybridization. The MMP-2 mRNA-related signal was stronger in patients with active MM (panel C) than in those with non-active MM (panel E) and with MGUS (panel G). The MMP-9 mRNA-related signal was lower than that of MMP-2 in all patient groups and overlapped between the groups (panels B, D, F). According to *in situ* hybridization results, SDS-PAGE gelatin-zymography of plasma cell CM (Fig. 4) showed that cells secrete activated MMP-2 (62 kDa form) and lower levels of activated MMP-9 (88 kDa form), whereas those from control subjects secrete only marginal levels of activated MMP-2 (not shown). Specifically, quantitation of MMP-2 and MMP-9 by computerized image analysis of the gelatinolytic bands showed that MMP-2 was present in CM of 84% active MM patients at $22.5 \pm 7.4 \times 10^3$ optical density (O.D.), compared with 27% non-active MM patients at $12.4 \pm 3.6 \times 10^3$ O.D., 25% MGUS patients at $13.4 \pm 5.4 \times 10^3$ O.D., and 16% control subjects at $4.0 \pm 1.7 \times 10^3$ O.D. MMP-9 was co-secreted in 23% active MM patients, 16% non-active MM patients and 10% MGUS patients at lower levels than MMP-2 and at overlapping levels between patient groups.

Overall results confirm the findings obtained in the MM cell line U266 and demostrate that in MM patients MMP-2 is overexpressed and secreted in step with tumor progression.

### MMP-2 and MMP-9 Expression by Mycosis Fungoides

To assess the ability of tumor T-cells to invade the ECM through the production of MMPs, we studied 57 biopsies of mycosis fungoides (MF) divided into three groups, i.e. patch, plaque and nodular (most advanced) groups, in accordance with histopathological diagnostic criteria for the disease (Willemze et al., 1994). The patch stage includes tumors formed by clustered lymphoid tumor T-cells within epidermis

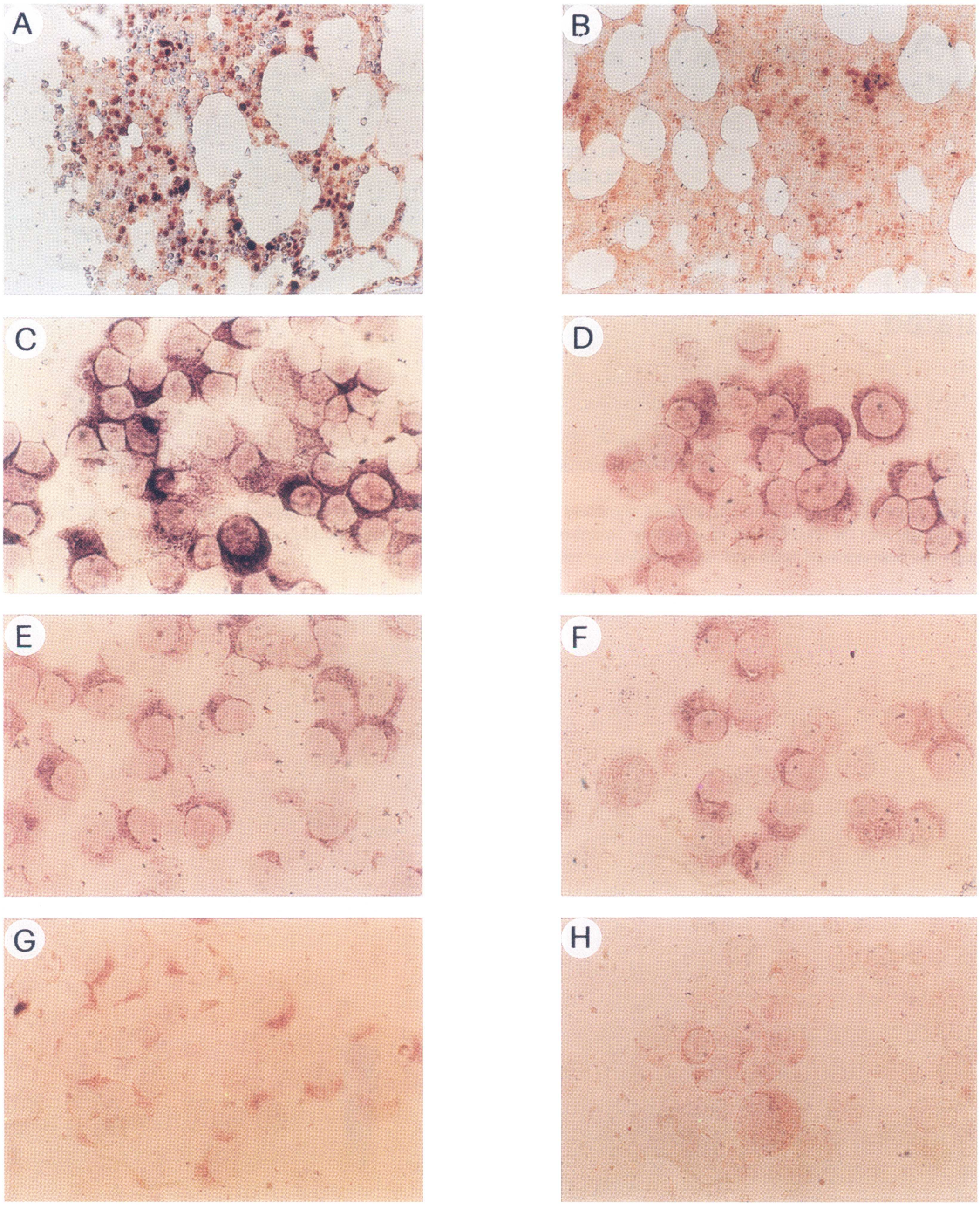

FIGURE 3 (A), (B) Bone marrow sections from a patient with active MM (relapse), and enriched bone marrow plasma cells isolated from patients with (C), (D) active MM (same patients as in [A], [B]), (E), (F) non-active MM (complete remission), (G) MGUS, and (H) from a control subject with pernicious anemia, tested with *in situ* hybridization for MMP-2 mRNA and MMP-9 mRNA. Note (A) numerous scattered plasma cells reactive for MMP-2 mRNA, whereas (B) fewer plasma cells were reactive for MMP-9 mRNA in the bone marrow of the active MM patient. These isolated and cytocentrifuged plasma cells were (C) strongly reactive for MMP-2 mRNA and (D) less reactive for MMP-9 mRNA. Also note (E) the weaker MMP-2 mRNA expression and (F) MMP-9 mRNA expression in the non-active MM patient, (G) the weak expression of MMP-2 mRNA in the MGUS patient and (H) no expression of MMP-2 mRNA in the control subject. Original magnifications: (A), (B), 125x, (C)-(H) 750x (see Color Plate VI at the back of this issue)

and superficial dermis, whereas the plaque stage refers to clustered and scattered cells within the epidermis and dermis, and nodular stage to clustered and scattered cells within epidermis and dermis in depth. The S-phase tumor growth fraction rises in the transition from one step to the next (Willemze et al., 1994).

The percentages of MF lesions expressing MMP-2 or MMP-9 mRNA and co-expressing both mRNAs increased in parallel with tumor progression (Table II). Compared to the patch stage, the plaque and even more the nodular stages gave significantly higher percentages of lesions expressing the MMP-2 mRNA ($p<0.05$ by chi-squared test), the MMP-9 mRNA ($p<0.05$) and both mRNAs ($p<0.05$).

The intensity of expression of MMP-2 and MMP-9 mRNA was assessed by the following score sistem: 1 (weak intensity), 2 (moderate), 3 (strong). The frequency of tumors scoring 1 or 2 remained fairly constant in all stages. By contrast, score 3 for both mRNAs occurred with significantly higher frequency in relation with tumor upstaging. For MMP-2 mRNA, it was detected in 18% of patch stage tumors, and in 31% and 52% of plaque and nodular stage tumors respectively ($p<0.05$). For MMP-9, score 3 was absent in the patch stage, whereas it was found in 13% and 31% of the plaque and nodular stage tumors respectively ($p<0.05$).

Histologically, the MMP-2 and MMP-9 mRNAs produced a cytoplasmic staining pattern (Fig. 5). In all MF stages, the mRNAs were not expressed homogeneously by the entire tumor cell population, but they were expressed by single cells or nests randomly scattered in the tumor area, which gave a very heterogeneous "ground-glass-like" staining pattern (panel A-D). These RNAs were generally not expressed by identical subpopulations of tumor cells. The location of positive cells was independent of the skin structures, such as hair follicles, vessels or glands, or of the skin layer (panel C).

In all stages, the mRNAs were also found in some stromal cell populations: MMP-2 mRNA in endothelial cells of intratumor and peritumor vessels and in fibroblasts, especially located in the stroma within or around the tumor clusters (panel E), MMP-9 in a subpopulation of tissue macrophages (identified with CD68 staining) frequently associated to tumor clusters. In contrast to MF lesions, the normal skin used as control expressed the MMP-2 mRNA in few microvascular endothelial cells and in none of the MMP-9 mRNA.

### DISCUSSION

Solid tumors are able to invade the ECM by means of matrix-degrading enzymes such as metalloproteinases and serine-proteases (Johnson et al., 1998). Here we report that lymphoid tumor B-cells and T-cells display similar capacity related to the production of MMP-2 and, to a lesser extent, of MMP-9.

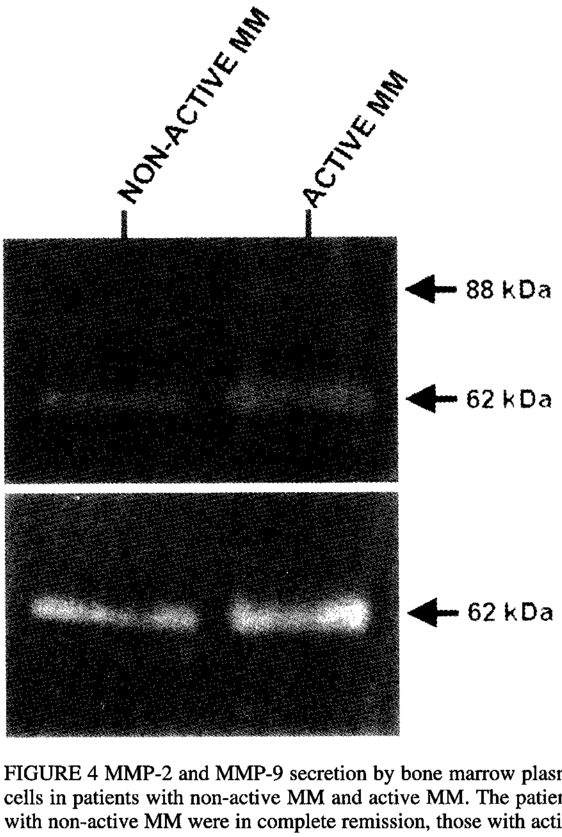

FIGURE 4 MMP-2 and MMP-9 secretion by bone marrow plasma cells in patients with non-active MM and active MM. The patients with non-active MM were in complete remission, those with active MM were at relapse (top) and at diagnosis (bottom). SDS-PAGE gelatin zymography of plasma cell CM sample. Note the bands of 62 kDa and 88 kDa corresponding to the activated form of MMP-2 and MMP-9 respectively. More intense MMP-2-related activity and MMP-9-related activity are evident in the active MM patients. The MMP-2-related activity is the highest

TABLE II Percent of positive lesions for the expression of MMP-2 and MMP-9 mRNA in mycosis fungoides

| *mRNA of* | *Stage* | | |
|---|---|---|---|
| | *Patch* *n = 16* | *Plaque* *n = 22* | *Nodule* *n = 19* |
| MMP-2 | 37.5[a] | 59.1 | 78.9 |
| MMP-9 | 12.5 | 31.8 | 52.6 |
| MMP-2 and MMP-9 | 6.3 | 22.7 | 42.6 |

a. Data expressed as percentages of lesions.

In human lymphoid tumor cell lines SB (B-cell lymphoblastic leukemia), U266 (multiple myeloma), CEM and Jurkat (T-cell lymphoblastic leukemia), the expression of MMP-2 and MMP-9 was demonstrated at the mRNA level through *in situ* hybridization, while the constitutive secretion of the enzymes was detected in the CM through the demonstration of the specific gelatinolytic activity. Other forms of human blood cancer, such as granulocytic sarcoma (Kobayashi et al., 1995), myeloid leukemia (Ries et al., 1994) and Burkitt's lymphoma (Kossakowska et al., 1996; Vacca et al., 1998) are able to secrete MMPs costitutively or after stimulation with phorbol ester (Ries et al, 1994; Watanabe et al, 1997).

Both MMPs are secreted in their activated, cleaved form. In fact, MMP-2 has an Mr of 72 kDa and MMP-9 an Mr of 92 kDa (Mignatti and Rifkin, 1993), while we found the 62 kDa and 88 kDa cleaved forms respectively in the CM. It is likely that, having been secreted as proenzymes, MMPs are rapidly cleaved at the cellular membrane level by the membrane-type MT-MMP (Butler et al, 1997), with which lymphoid tumor cells could be equipped. Since the lymphoblastoid cells secrete activated MMP-2 and MMP-9, as well as urokinase-type plasminogen activator (uPA) (Mignatti and Rifkin, 1993), and since these enzymes lyse the collagens and fibronectin, which are subendothelial basement membrane and interstitial stroma ECM constituents, it is conceivable that these cells are capable of invading the stroma and the vessel wall and, partly by this way, are liable to enter the circulation. This would enable them to enter distant body sites via the same mechanisms.

As observed for tumor cells from invasive and metastatic solid tumors (Weidner, 1998), the bone marrow plasma cells of active MM patients express and secrete high levels of MMP-2 (Vacca et al., 1999). In some patients, sizeable levels of MMP-9 were co-expressed and co-secreted. As the progression from *in situ* to invasive and metastatic solid tumors is accompanied and enhanced by secretion of proteolytic enzymes (Johnson et al., 1998), our findings suggest that active MM may represent the invasive phase of plasma cell tumors. Typically, the plasma cell MMPs were observed in the CM in their cleaved form. Also for tumor plasma cells, it is likely that the secreted MMPs are rapidly cleaved and activated by the MT-MMP system on the cell surface.

Others (Barillé et al., 1997) have shown production of MMP-9 by an array of MM cell lines (prominently by RPMI-8226 cell line) and freshly isolated bone marrow plasma cells of MM patients, though no correlation with disease activity has been found. It has also been shown that bone marrow stromal cells (i.e. fibroblasts and osteoblasts) of MM patients produce constitutively MMP-1 and the MMP-2 proenzyme which is converted into active MMP-2 in co-cultures with MM plasma cells (Barillé et al., 1997). Furthermore, IL-1$\beta$ and TNF-$\alpha$, i.e. cytokines produced by MM plasma cells and stromal cells respectively (Merico et al., 1993; Klein, 1995), enhance MMP-1 production. Still other studies describe the stimulating role of IL-1$\beta$ on the production of MMPs by bone marrow stromal cells (Guedez et al., 1996).

Hence, in addition to the production of MMP-2, which is greater in active MM, and of MMP-9 by the MM plasma cells, there is a production of MMP-1 and MMP-2 by the bone marrow stromal cells stimulated (via IL-1$\beta$?) by the plasma cells themselves. Given the ability of MMP-1 to degrade type I collagen and of MMP-2 and MMP-9 to degrade type IV, V, VII and X collagens as well as fibronectin (Mignatti and Rifkin, 1993; Johnson et al, 1998), the data suggest that plasma cells of active MM patients are especially capable of invading both the stroma and the basement membrane of the ECM. In addition, the degradation of ECM brings about the release of angiogenic factors that are stored inside it (Mignatti and

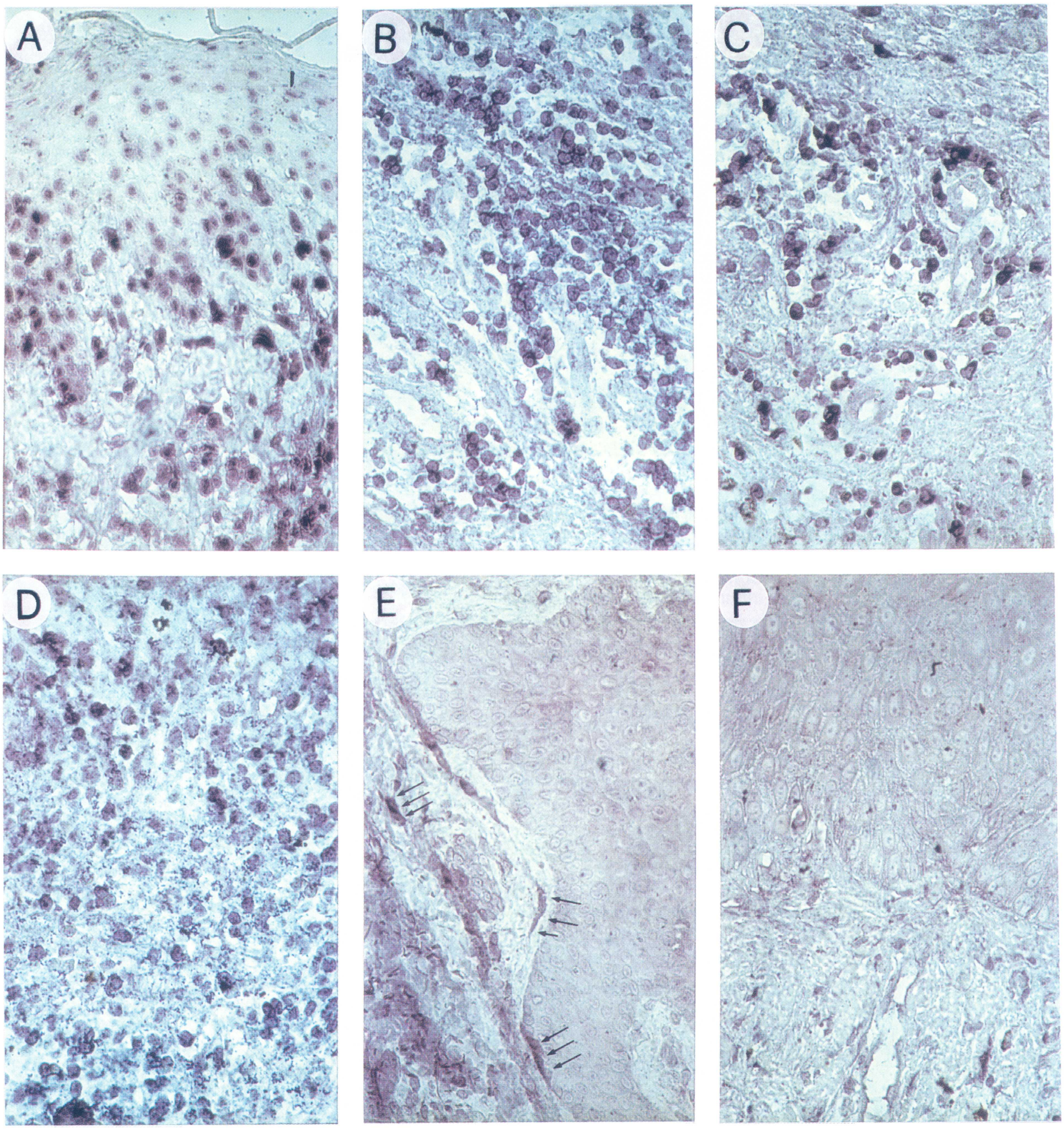

FIGURE 5 Mycosis fungoides: (A), (F) plaque stage (B)-(E) nodular stage, tested by *in situ* hybridization for (A)-(C), (E) MMP-2 mRNA and (D) MMP-9 mRNA. The colorimetric signal is present in most tumor cells both in the plaque and nodular stages. Note sub (A) scattered tumor cells in superficial dermis and epidermis, sub (B)-(D) clustered tumor cells in the deep dermis and sub (C) around vascular structures. The signal produces a "ground-glass-like" picture, expecially sub (D). Note sub (E) intense signal for MMP-2 mRNA in spindle-shaped fibroblasts (arrows) within the tumor cells. The negative control for MMP-2 is sub (F) in an adjacent section. Original magnifications: (A)-(F) 300x (see Color Plate VII at the back of this issue)

Rifkin, 1993), though plasma cells are able to secrete angiogenic factors in active MM (Vacca et al., 1999). Thus, overall data suggest that major proteolytic activity and angiogenesis occur together in close vicinity of plasma cells, and that conditions of intra- and extramedullary disseminations of the plasma cells themselves are thereby established.

Along with MM plasma cells, malignant B cells in non-Hodgkin's lymphomas have been shown to be an important source of MMP-2, MMP-9 (Stetler-Stevenson et al., 1997; Kossakowska et al., 1998) and uPA (Philip-Joet et al., 1995). The proteolytic activity connected with such enzymes is thought to be responsible for the extensive permeation of organs, with destruction of their anatomical boundaries in a fashion similar to that observed in carcinomas (Kossakowska et al., 1996; 1998).

In MF, the production of MMPs increases in step with progression stages. MMP-2 and MMP-9 mRNAs are significantly upregulated from patch to nodular stage, as indicated by more frequent tumors expressing one or both mRNAs and more frequent tumors showing the strongest expression intensity in the nodular stage. Thus, both MMPs are produced more frequently and in greater quantities with advancing MF progression, suggesting that a more intense degradation of interstitial stroma and subendothelial basement membrane takes place with progression. This may explain the greater dissemination and deepening of MF cells into dermis as the disease progresses from the patch to the nodular stage, and accounts, together with denser angiogenesis in the plaque and even more in the nodular stage (Vacca et al., 1997), for frequent spreading of MF cells in lymph nodes, and parenchymal organs during these stages. Finally, MMP-2 mRNA was detected in microvascular endothelial cells and fibroblasts, whereas MMP-9 mRNA was seen in a subset of macrophages. By contrast, in normal skin only MMP-2 mRNA was expressed by few microvascular endothelial cells. Thus, the expression of MMPs by stromal cells characterize the malignant condition, and agree with findings in solid tumours (Polverini, 1996; Leek et al., 1997; Ribatti et al., 1999). Stromal cells, probably recruited and activated by MF cells, produce the MMPs, thus participating in the degradation of the extracellular matrix and contributing to the tumor dissemination. Therefore, regulation of ECM degradation during tumor progression is the result of a concerted action, not only of several proteolytic systems, but also of several cell types, including both malignant and non-malignant cells in tumor stroma.

Overall, our results may explain the frequent occurrence of extranodal or extramedullary localizations in human lymphoproliferative diseases, and suggest that combination therapy with protease inhibitors plus cytotoxic drugs successfully applied in animal solid tumors (Folkman, 1996; Stetler-Stevenson, 1999) could perhaps be considered in these diseases.

### MATERIAL AND METHODS

### Cell Lines and Preparation of Conditioned Medium (CM)

SB, CEM, Jurkat and U266 cell lines were obtained from the American Type Culture Collection (Rockville, MD, USA) and grown in RPMI-1640 medium supplemented with 10% heat inactivated calf serum (FCS) and 1% glutamine at 37°C in 5% $CO_2$ humidified atmosphere. After culturing for 24 h in medium without FCS (serum free medium – SFM), the conditioned medium (CM) was collected, sequentially centrifuged at 1,200 and 12,000 rpm for 10 min respectively, filtered through sterilized 0.22 μm pore-size filters (Costar, Cambridge, MA), and stored at –80°C.

### Bone Marrow Plasma Cell Cultures

Aspirates were subjected to Ficoll-Hypaque density gradient centrifugation and plasma cell enrichment. T cells were removed by two-fold E-rosetting, monocytes and fibroblasts by adhesion determined by culturing in plastic flasks for 90 min in RPMI-1640 containing 10% FCS at 37°C in 5% $CO_2$ humidified atmosphere. Residual cells were incubated with magnetic beads (Dynal, Oslo, Norway) coated with antibody to the plasma cell marker CD38 (Becton & Dickinson, Mountain View, CA). After magnetic subtraction and bead detachment by culturing cells for 12 h in RPMI-1640 supplement d with 10% FCS at 37°C in 5% $CO_2$ humidified atmosphere, enriched plasma cells were obtained. These cells contained <2% of T cells and monocytes, as assessed by flow cytometry and with the anti-CD3 and anti-CD68 antibodies respectively (FACScan, Becton & Dickinson) and consisted of >95% tumor plasma cells and their clonally-related cells (Billadeau et al., 1993), as assessed by morphology with May-Grünwald-Giemsa and flow cytometry with the anti-CD38 antibody (Becton & Dickinson). Cells were cultured ($1 \times 10^7$ per 25 $cm^2$ flask) for 24 h in SFM, and their viability assessed by trypan blue exclusion was >90%. The plasma cell CM was prepared as described above.

### MMP-2 and MMP-9 sodium dodecyl sulfate (SDS)-polyacrylamide gel electrophoresis (PAGE) gelatin-zymography

SDS-PAGE gelatin-zymography of CM was performed to visualize the gelatinolytic activity of the secreted MMP-2 and MMP-9 (Albini et al., 1987). Ten µg of CM proteins were applied to 7.5% SDS-PAGE gels co-polymerized with type A gelatin from porcine skin (Sigma Chemical Co., St. Louis, MO) at a final concentration of 0.6 mg/ml. After electrophoresis, gels were washed in 2.5% Triton 1x for 1 h to remove SDS, incubated for 18 h at 37°C and stained in 0.1% Coomassie brilliant blue. The gelatinolytic regions were observed as white bands against a blue background. The levels of MMP activity were assessed by scoring the intensity of the bands by a computerized image analysis (APPLE, Cupertino, CA).

### In Situ Hybridization of MMP-2 and MMP-9 mRNA

This was performed as described previously (Vacca et al., 1997; 1998). Cytocentrifuged plasma cells ($1–2 \times 10^5$ per slide) were fixed in 4% paraformaldehyde, washed in phosphate-buffered saline (PBS), made permeable with 10 µg/ml of proteinase K (Sigma Chemical Co.) in $CaCl_2$ (2 mM)-tris(hydroxymethyl)aminomethane (TRIS) (20 mM) for 5 min at 37°C, acetylated with 0.25% acetate in 0.1 M triethanolamine, washed in 2x standard saline citrate (SSC), dehydrated in graded ethanols and air-dried. Cells were hybridized overnight at 50°C with 5 µg/ml of two 5′-biotinylated oligonucleotides (Genenco Life Sciences, Florence, Italy), the first of 42 bases complementary to the ninth exon sequence 446–459 of the MMP-2 mRNA, the second of 48 bases complementary to the ninth exon sequence 445–460 of the MMP-9 mRNA (Huhtala et al., 1991). Fifty percent deionized formamide, 600 mM NaCl, 80 mM TRIS, 4 mM EDTA, 10 mM dithiothreitol, 1x Denhardt's solution and 100 µg/ml salmon sperm DNA were used as hybridization medium. After washing in 2x to 0.01x SSC and in PBS, cytospins were incubated overnight at 4°C with streptavidin-alkaline phosphatase conjugate (Promega Co., Madison, WI). After alkaline phosphatase activity was revealed by Western blue stabilized substrate (Promega Co.), cytospins were mounted in buffered glycerin and evaluated by two observers with a double-headed Leitz Dialux 20 photomicroscope (Leitz, Wetzlar, Germany). Cells were scored positive for the hybridization signal relative to the background signal of RNAse-treated control cytospins hybridized with the same oligonucleotides as follows: (-), no signal; (+), detectable signal; (2+), easily visualized signal. For MF tissues, deparaffinized sections processed as described above were used. After detection of alkaline phosphatase activity by Western blue stabilized substrate (Promega Co.), sections were mounted in buffered glycerine, and then evaluated by two investigators under the photomicroscope for the extent and intensity of the hybridization signal within the tumor area. The signal was scored relative to the background signal of RNAse-treated sections hybridized with the same oligonucleotides (negative controls) as follows: (1) weak (detectable), (2) moderate (easily visualised) or (3) strong. MF were considered positive when >10% of tumor cells gave the signal. Negative MF (scored as 0) were those with ≤10% of tumor cells displaying the signal. The cut-off was based on the finding that ≤10% of tumor cells seldom gave the signal in negative controls.

### Acknowledgements

This study was supported in part by the Associazione Italiana per la Ricerca sul Cancro (A.I.R.C.), Milan, and Ministry of Education (M.U.R.S.T. – co-financed and C03 funds), Rome Italy. Mr. Roberto Gabrieli is gratefully acknowledged for preparing the figures.

### References

Albini A., Iwamoto Y., Kleinman H.K., Martin G.R., Aaronson S.A., Kozlowski J.M., McEvan R.N. (1987). A rapid *in vitro* assay for quantitating the invasive potential of tumor cells. Cancer Res. 47: 3239–3245.

Amorino G.P., Hoover R.L. (1998). Interactions of monocytic cells with human endothelial cells stimulate monocytic metalloproteinase production. Am. J. Pathol. 152: 199–207.

Barillé S., Akhoundi C., Collette M., Mellerin M.-P., Rapp M.-J., Harousseau J.-L., Bataille R., Amiot M. (1997). Metalloproteinases in multiple myeloma: production of matrix metalloproteinase-9 (MMP-9), activation of proMMP-2, and induction of MMP-1 by myeloma cells. Blood 90: 1649–1655.

Billadeau D., Ahmann G., Greipp P., Van Ness B. (1993). The bone marrow of multiple myeloma patients contains B cell populations at different stages of differentiation that are clonally related to malignant plasma cell. J. Exp. Med. 178: 1023–1031.

Bottomley K.M., Johnson W.H., Walter D.S. (1998). Matrix metalloproteinase inhibitors in arthritis. J. Enzyme Inhib. 13: 79–101.

Butler G.S., Will H., Atkinson S.J., Murphy G. (1997). Membrane-type 2 matrix metalloproteinase can initiate the processing of progelatinase A and is regulated by the tissue inhibitors of metalloproteinases. Eur. J. Biochem. 244: 653–657.

Coussens L.M., Werb Z. (1996). Matrix metalloproteinases and the development of cancer. Chem. Biol. 3: 895–904.

Di Girolamo N., Tedla N., Lloyd A., Wakefield D. (1998). Expression of matrix metalloproteinases by human plasma cells and B lymphocytes. Eur. J. Immunol. 28: 1773–1784.

Folkman J. (1995). Angiogenesis in cancer, vascular, rheumatoid and other disease. Nature Med. 1: 27–31.

Folkman J. (1996). New perspectives in clinical oncology from angiogenic research. Eur. J. Cancer 32A: 2534–2539.

Gordon J.L., Drummond A.H., Galloway W.A. (1993). Metalloproteinase inhibition as therapeutics. Clin. Exp. Rheumatol. 11: S91–94.

Guedez L., Lim M.S., Stetler-Stevenson W.G. (1996). The role of metalloproteinases and their inhibitors in hematologic disorders. Crit. Rev. Oncogen. 7: 205–225.

Huhtala P., Tuuttila A., Chow L.T., Lohi J., Keski-Oja J., Tryggvason K. (1991). Complete structure of the human gene for 92-kDa type IV collagenase. Divergent regulation of expression for the 92- and 72-kDa enzyme genes in HT-1080 cells. J. Biol. Chem. 266: 16485–16490.

Johnson L.L., Dyer R., Hupe D.J. (1998). Matrix metalloproteinases. Curr. Opin. Chem. Biol. 4: 466–471.

Kanayama H., Yokota K., Kurokawa H., Murakami Y., Nishitani M., Kagawa S. (1998). Prognostic values of matrix metalloproteinase-2 expression in bladder cancer. Cancer 82: 1359–1366.

Klein B. (1995). Cytokine, cytokine receptors, transduction signals and oncogenes in multiple myeloma. Sem. Hematol. 32: 4–19.

Kobayashi M., Hamada J., Li Y.-Q., Shinobu N., Imamura M., Okada F., Takeichi N., Hosokawa M. (1995). A possible role of 92 kDa type IV collagenase in the extramedullary tumor formation in leukemia. Jpn. J. Cancer Res. 86: 298–303.

Kossakowska A.E., Hinek A., Edwards D.R., Lim M.S., Zhang C.-L., Breitman D.R., Prusinkiewicz C., Stabbler A.L., Urbanski L.S., Urbanski S.J. (1998). Proteolytic activity of human non-Hodgkin's lymphoma. Am. J. Pathol. 152: 565–576.

Kossakowska A.E., Hucheroft S.A., Urbanski S.J., Edwards D.R. (1996). Comparative analysis of the expression patterns of metalloproteinases and their inhibitors in breast neoplasia, sporadic colorectal neoplasia, pulmonary carcinomas and malignant non-Hodgkin's lymphomas in humans. Br. J. Cancer 73: 1401–1408.

Krane S.M. (1994). Clinical importance of metalloproteinases and their inhibitors. Ann. NY Acad. Sci. 732: 1–10.

Leek R.D., Lewis C.E., Harris A.L. (1997). The role of macrophages in tumor angiogenesis. In Tumor Angiogenesis, Bicknell R., Lewis C.E., Ferrara N. Eds. (Oxford: Oxford University Press), pp. 81–89.

Machein U., Conca W. (1997). Expression of several matrix metalloproteinase genes in human monocytic cells. Adv. Exp. Med. Biol. 421: 247–251.

Madri J.A., Graesser D., Haas T. (1996). The roles of adhesion molecules and proteinases in lymphocyte transendothelial migration. Biochem. Cell. Biol. 74: 749–757.

Massova I., Kotra L.P., Fridman R., Mobashery S. (1998). Matrix metalloproteinases: structures, evolution and diversification. FASEB J. 12: 1075–1095.

Merico F., Bergui L., Gregoretti M.G., Ghia P., Aimo G., Lindley I.J.D., Caligaris-Cappio F. (1993). Cytokines involved in the progression of multiple myeloma. Clin. Exp. Immunol. 92: 27–31.

Mignatti P., Rifkin D.B. (1993). Biology and biochemistry of proteinases in tumor invasion. Physiological Rev. 73: 161–195.

Philip-Joet F., Alessi M.C., Philip-Joet C., Aillaud M., Barriere J.R., Arnaud A., Juhan-Vague I. (1995). Fibrinolytic and inflammatory process in pleural effusions. Eur. Resp. J. 8: 1352–1356.

Polverini P.F. (1996). How the extracellular matrix and macrophages contribute to angiogenesis-dependent diseases. Eur. J. Cancer 32A: 2430–2437.

Ribatti D., Vacca A., Nico B., Quondamatteo F., Ria R., Minischetti M., Marzullo A., Herken R., Roncali L., Dammacco F. (1999). Bone marrow angiogenesis and mast cell density increase simultaneously with progression of human multiple myeloma. Br. J. Cancer 79: 451–455.

Ries C., Kalb H., Petrides P.E. (1994). Regulation of 92 kD gelatinase release in HL-60 leukemia cells: Tumor necrosis factor-α as an autocrine stimulus for basal- and phorbol ester-induced secretion. Blood 83: 3638–3646.

Stetler-Stevenson W.G. (1999). Matrix metalloproteinase and angiogenesis: a moving target for therapeutic intervention. J. Clin. Invest. 103: 1237–1241.

Stetler-Stevenson M., Mansoor A., Lim M., Fukushima P., Kehrl J., Marti G., Ptaszynski K., Wang J., Stetler-Stevenson W.G. (1997). Expression of matrix metalloproteinases and tissue inhibitors of metalloproteinases in reactive and neoplastic lymphoid cells. Blood 89: 1708–1715.

Trocme C., Gaudin P., Berthier S., Barro C., Zaoui P., Morel F. (1998). Human B lymphocytes synthesize the 92 kDa type IV gelatinase, matrix metalloproteinase-9. J. Biol. Chem. 273: 20677–20684.

Vacca A., Moretti S., Ribatti D., Pellegrino A., Pimpinelli N., Bianchi B., Bonifazi E., Ria R., Serio G., Dammacco F. (1997). Progression of mycosis fungoides is associated with changes in angiogenesis and expression of the matrix metalloproteinase-2 and –9. Eur. J. Cancer 33: 1685–1692.

Vacca A., Ribatti D., Iurlaro M., Albini A., Minischetti M., Bussolino F., Pellegrino A., Ria R., Rusnati M., Presta M., Vincenti V., Persico M.G., Dammacco F. (1998). Human lymphoblastoid cells produce extracellular matrix-degrading enzymes and induce endothelial cell proliferation, migration, morphogenesis and angiogenesis. Int. J. Clin. Lab. Res. 28: 55–68.

Vacca A., Ribatti D., Presta M., Minischetti M., Iurlaro M., Ria R., Albini A., Bussolino F., Dammacco F. (1999). Bone marrow neovascularization, plasma cell angiogenic potential and matrix metalloproteinase-2 secretion parallel progression of human multiple myeloma. Blood 93: 3064–3073.

Watanabe K., McCormick K.L., Volker K., Ortaldo J.R., Wiggington J.M., Brunda M.J., Wiltrout R.H., Fogler W.E. (1997). Regulation of host-mediated anti-tumor mechanisms of cytokines. Direct and indirect effects on leukocyte recruitment and angiogenesis. Am. J. Pathol. 150: 1869–1880.

Weeks B.S., Schnaper H.W., Handy M., Holloway E., Kleinman H.K. (1993). Human T lymphocytes synthesize the 92 kDa type IV collagenase (gelatinase B). J. Cell. Physiol. 157: 644–649.

Weidner N. (1998). Tumoral vascularity as a prognostic factor in cancer patients: the evidence continues to grow. J. Pathol: 184: 119–122.

Willemze R., Beljaards R.C., Meijer C.J.L.M. (1994). Classification of primary cutaneous T-cell lymphomas. Histopathology 24: 405–415.

